# Empyema Thoracis from *Salmonella* Choleraesuis

**DOI:** 10.3201/eid1109.050030

**Published:** 2005-09

**Authors:** Chih-Cheng Lai, Li-Na Lee, Po-Ren Hsueh, Chong-Jen Yu, Pan-Chyr Yang

**Affiliations:** * National Taiwan University Hospital, Taipei, Taiwan, Republic of China

**Keywords:** nontyphoid *Salmonella*, *Salmonella enterica* serotype Choleraesuis, empyema thoracis, letter

**To the Editor:** The clinical presentations of nontyphoidal *Salmonella* infection are protean, including gastroenteritis (most common), bacteremia, septic arthritis, osteomyelitis, and endovascular infection (*1*). Despite the growing number of patients with invasive infection due to nontyphoidal *Salmonella*, reports of thoracic empyema due to these organisms remain rare (*2**–**6*).

We searched the computer database of our microbiology laboratory for patients with positive pleural effusion culture from June 1997 to February 2004. Patients were included if they met the following criteria: 1) thoracentesis recovered purulent pleural fluid; 2) microorganisms identified by microscopic examination; and 3) a *Salmonella* species isolated from a pleural effusion specimen.

Isolates of *Salmonella* were identified to the serotype level, according to the Kauffman and White scheme, using somatic and flagellar antigens (Denka Seiken Co., Ltd., Tokyo, Japan) and also by conventional methods and the Phoenix System (panel type, NMIC/ID4) (Becton Dickson, Sparks, MD, USA) (*7*). Susceptibilities of *Salmonella* isolates to ampicillin, cefotaxime, chloramphenicol, ciprofloxacin, and trimethoprim-sulfamethoxazole were determined by the disk diffusion method. Organisms were categorized as susceptible or resistant to the antimicrobial agents tested on the basis of National Committee for Clinical Laboratory Standards (NCCLS) guidelines (*8*). Antimicrobial therapy was considered to be appropriate when the antimicrobial agent was active in vitro by the disk diffusion susceptibility method against a *Salmonella* isolate.

During the study, 973 patients with a diagnosis of empyema thoracis were identified; 12 (1.23%) of these patients, including 9 men and 3 women, were infected with *Salmonella* species. The clinical characteristics of the 12 patients are summarized in the Table A1. The median age was 49 years; 1 patient was >65 years of age. Underlying diseases were present in all patients, including 7 with malignancy, 5 with gallstones, and 3 each with diabetes mellitus and chronic renal failure. Five patients had used antacids and 3 patients had received chemotherapy or steroids. Ten patients (83.3%) were immunocompromised and had a variety of illnesses, including malignancy, liver cirrhosis, and diabetes mellitus. Common symptoms were dyspnea (83.3%), fever (75%), and cough (50%). Analysis of pleural effusion showed a median leukocyte count of 25,600/μL, a lactate dehydrogenase level of 513 U/L, and a glucose level of 88 mg/dL. Gram staining was conducted on 3 patients' pleural effusion but none of them showed positive results.

Twenty-three *Salmonella* isolates were recovered as the sole pathogen from various clinical specimens, including pleural effusion (15 isolates), blood (6 isolates), ascites (1 isolate), and aortic wall (1 isolate). Among the 12 patients with empyema thoracis, 4 had *Salmonella enterica* serotype Typhimurium (*S*. Typhimurium) and 1 had group C2 *Salmonella* during 1997–1999; 7 patients had *Salmonella enterica* serotype Choleraesuis (*S*. Choleraesuis) after 1998. All *S*. Typhimurium and group C2 *Salmonella* were isolated from pleural effusion specimens, but *S*. Choleraesuis was isolated from multiple extrapulmonary sites including blood, ascites, and aortic wall (Table A1). Although the number of study cases is limited, it may suggest that *S*. Choleraesuis is more invasive than 2 other *Salmonella* species.

Among the *S*. Choleraesuis isolates recovered from 7 patients, 2 were resistant to ampicillin and sulfamethoxazole-trimethoprim, 3 were resistant to chloramphenicol, 1 was resistant to ciprofloxacin, and all were susceptible to cefotaxime. All *S*. Typhimurium isolates were susceptible to sulfamethoxazole-trimethoprim, ciprofloxacin, and cefotaxime. Two of the 4 patients had isolates that were resistant to chloramphenicol, and 2 other patients had isolates that were resistant to ampicillin. The group C2 *salmonella* isolate was resistant to chloramphenicol only.

Among the 12 *Salmonella* isolates from patients with empyema thoracis, 9 were resistant to >1 commonly used antimicrobial. Treatment and outcome information was available for 11 of the 12 patients. All 11 patients received antimicrobials drugs (median duration 35 days); this therapy was appropriate in 9 of 11 patients. Six patients had thoracentesis, 2 had close tube thoracostomy, and 1 had open drainage. One of the 4 patients with *S*. Typhimurium empyema who did not receive appropriate antimicrobial drugs died. In contrast, 4 (57%) of the 7 patients with *S*. Choleraesuis infection, including one who did not receive appropriate antimicrobial therapy, died. Another factor related to outcome was drainage. One (20%) of the 5 patients who underwent tube thoracostomy or thoracoscopy died, while 3 (50%) of the 6 patients who underwent thoracentesis died. All 3 of these patients had *S*. Choleraesuis.

Most (92%) of our patients were <65 years of age. These data indicate that *Salmonella* should be considered as a potential cause of thoracic empyema, even in younger patients, especially in the presence of malignancy or hepatobilliary disease. More than half of our patients had used antacids or had suffered from gallstones. This finding suggests that susceptibility to *Salmonella* infection may be increased by alterations in the gastrointestinal tract, including decreased gastric acidity and chronic gastrointestinal disease. Leukocytosis was noted in 25% of patients. In fact, two thirds of the patients had a normal leukocyte count with immature leukocytes, which may be attributable to their relatively impaired cell-mediated immunity.

The predominant organism in this series was *S*. Choleraesuis, followed by *S*. Typhimurium. In Taiwan, the rate of resistance of *S*. Choleraesuis to ampicillin, chloramphenicol, or sulfamethoxazole-trimethoprim increased to approximately 90% for all 3 drugs and the rate of resistance to ciprofloxacin was from 7.7% to 59% (*5**–**7*). The resistance rate of *S*. Choleraesuis to ciprofloxacin in this study was similar to our previous report (*7*).

Nine of the 11 patients who completed follow-up information received appropriate antimicrobial drugs with drainage; however, 4 died. These 4 deaths (57%) were due to *S*. Choleraesuis-related empyema; 3 patients had underlying malignancy. Although appropriate antimicrobial drugs were used, our data suggest that more aggressive treatment with open drainage of the pleural effusion might have contributed to a better outcome than closed tube thoracostomy or simple thoracocentesis alone. In contrast to *S*. Choleraesuis-related infection, all 4 patients with non–*S*. Choleraesuis-related thoracic empyema survived. One of these patients did not receive appropriate antimicrobial drug treatment, but did have adequate drainage with simple thoracocentesis. This suggests adequate and aggressive drainage of pleural effusion may be as important as appropriate antimicrobial drugs. However, the overall death rate (36%) in this study was still higher than that of other reports (*9*). This might have been due to the high number of immunocompromised patients in this study.

In conclusion, thoracic empyema is a rare complication of nontyphoid *Salmonella* infection and is closely associated with an immunocompromised condition, even in patients <65 years of age. Higher rates of resistance and death were noted in patients with empyema thoracic caused by *S*. Choleraesuis. Early diagnosis, appropriate antimicrobial drug therapy, and aggressive drainage are necessary to improve the outcome of patients with thoracic empyema due to *S*. Choleraesuis.

## Appendix

**Table A1:** Demographic and clinical features of 12 patients with empyema thoracis due to non-typhoid *Salmonella**

Patient no.	Sex	Age, y	Underlying condition(s)	Use of antacids	Site(s) of isolation (no. of isolation)	Etiology	Manifestations	Type	Resistance profiles of antimicrobial drugs
1	M	39	NPC	-	Pleural effusion (1)	Aspiration pneumonia	Cough, fever, dyspnea	*Salmonella*, group C2	C
2	M	58	Thymic cancer, DM	+	Pleural effusion (1)	CAP	Fever, dyspnea, chest pain	*S*. Typhimurium	None
3	F	50	Breast cancer	-	Pleural effusion (1)	CAP	Cough, fever	*S*. Typhimurium	C
4	M	41	Liver cirrhosis, gall bladder stones	-	Pleural effusion (2)	CAP	Fever, dypsnea	*S*. Typhimurium	C, AM
5	M	49	Lung cancer	-	Pleural effusion (1)	Obstructive pneumonia	Cough, fever, dyspnea	*S*. Typhimurium	AM
6	M	38	Colon cancer	+	Pleural effusion (1)	CAP	Cough, dyspnea	*S*. Choleraesuis	None
7	F	48	Breast cancer, cervical cancer	+	Pleural effusion (1)	CAP	Dyspnea, chest pain	*S*. Choleraesuis	C, AM, SXT
8	F	67	DM, liver cirrhosis, gall bladder stones, CRF	+	Pleural effusion (1), blood (2), ascites	Spontaneous bacterial empyema	Fever, dyspnea, abdominal pain	*S*. Choleraesuis	None
9	M	30	Lung cancer	-	Pleural effusion (1), blood (2)	CAP	Cough, fever, dyspnea	*S*. Choleraesuis	C, GM
10	M	53	CRF, CAD, gall bladder stones	+	Pleural effusion (1), aortic wall	Mycotic aneurysm	Fever, abdominal pain	*S*. Choleraesuis	None
11	M	51	DM, liver cirrhosis, gall bladder stones, CRF	-	Pleural effusion (3), blood (2)	Obstructive pneumonitis	Cough, dyspnea	*S*. Choleraesuis	AM
12	M	49	Alcoholism, gall bladder stones, CAD	-	Pleural effusion (1)	CAP	Fever, dyspnea	*S*. Choleraesuis	C, AM, CIP, SXT

*NPC, nasopharyngeal cancer; C, chloramphenicol; DM, diabetes mellitus; CAP, community acquired pneumonia; AM, ampicillin; SXT, sulfamethoxazole-trimethoprim; CRF, chronic renal failure; GM, gentamicin; CAD, coronary artery disease; CIP, ciprofloxacin.
